# Supplementary material for: Kaempferol Inhibits Zearalenone-Induced Oxidative Stress and Apoptosis via the PI3K/Akt-Mediated Nrf2 Signaling Pathway: In Vitro and In Vivo Studies
Source: Int J Mol Sci. 2020 Dec 28;22(1):217. doi: 10.3390/ijms22010217 (PMC7794799; doi:10.3390/ijms22010217)
Supplement: Supplementary file 1 [file ijms-22-00217-s001.zip › ijms-1012156-supplementary pending conversion (1).docx]

Supplementary Materials

Kaempferol Inhibits Zearalenone-Induced
Oxidative Stress and Apoptosis
via the PI3K/AKT-Mediated Nrf2 Signaling Pathway:
In Vitro and In Vivo Studies

Peramaiyan Rajendran ^1,^*^,†^, Rebai Ben Ammar ^1,2^, Fatma J. Al-Saeedi ^3,†^, Maged E. Mohamed ^4,5^, Medhat A. ElNaggar ^6,7^, Saeed Y. Al-Ramadan ^8^, Gamal M. Bekhet ^1,9^ and Ahmed M. Soliman ^10,11^

^1^ Department of Biological Sciences, College of Science, King Faisal University, Al-Ahsa Post Box 31982, Saudi Arabia; rbenammar@kfu.edu.sa (R.B.A.); gbekhet@kfu.edu.sa (G.M.B.)

^2^ Laboratory of Aromatic and Medicinal Plants, Center of Biotechnology, Technopole of Borj-Cedria, Hammam-Lif PBOX 901 2050, Tunisia

^3^ Department of Nuclear Medicine, Faculty of Medicine, Kuwait University, Safat 13110, Kuwait; fatma.alsaeedi@ku.edu.kw

^4^ Pharmaceutical Sciences Department, College of Clinical Pharmacy, King Faisal University, Al-Ahsa 31982, Saudi Arabia; memohamed@kfu.edu.sa

^5^ Pharmacognosy Department, College of Pharmacy, Zagazig University, Zagazig 44519, Egypt

^6^ Plant Pathology Research Institute, Agricultural Research Center, Giza Governorate 1266, Egypt; drnaggar@sago.gov.sa

^7^ Research Central Laboratory, Saudi Grains Organization, Riyadh 12343, Saudi Arabia

^8^ Department of Anatomy, College of Veterinary Medicine, King Faisal University, Al-Ahsa 31982, Saudi Arabia; salramadan@kfu.edu.sa

^9^ Department of Zoology, Faculty of Science, Alexandria University Egypt; Alexandria 21544, Egypt

^10^ Department of Arid Land Agriculture, College of Agricultural & Food Sciences, King Faisal University, Al Ahsa 31982, Saudi Arabia; amohamed@kfu.edu.sa

^11^ Virus & Phytoplasma Research Department, Plant Pathology Research Institute, Agricultural Research Center, Giza Governorate 1266, Egypt

***** Correspondence: prajendran@kfu.edu.sa; Tel.: +966-5899543; Fax: +966-013-5899556

† These authors contributed equally to this work.


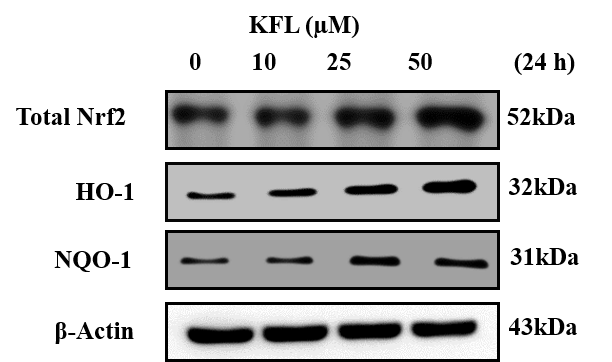


**Figure S1.** Effect of KFL on HepG2 cells.

**Table S1.** Effect of KFL with ZEA treated mice on Body and liver weight.


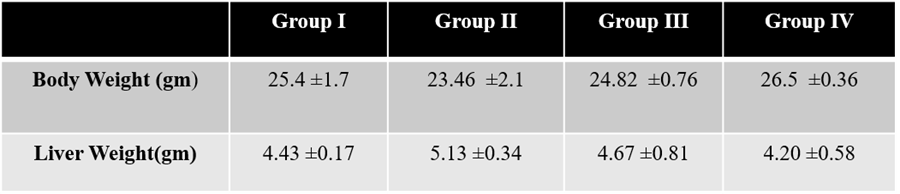


Control and treated animals did not showed significance variation.

**Video.** Animal behavioral Studies.

**Video 1.** Control group. All animals have shown normal behavior.

**Video 2.** ZEA alone treated. Animal has shown abnormal behavior, agitated and fight with others.

**Video 3.** ZEA alone treated. All animal have shown abnormal behavior.

**Video 4.** KFL with ZEA treated. All animals have shown normal behavior and activity.

**Video 5.** KFL alone treated. All animal have shown normal behavior.


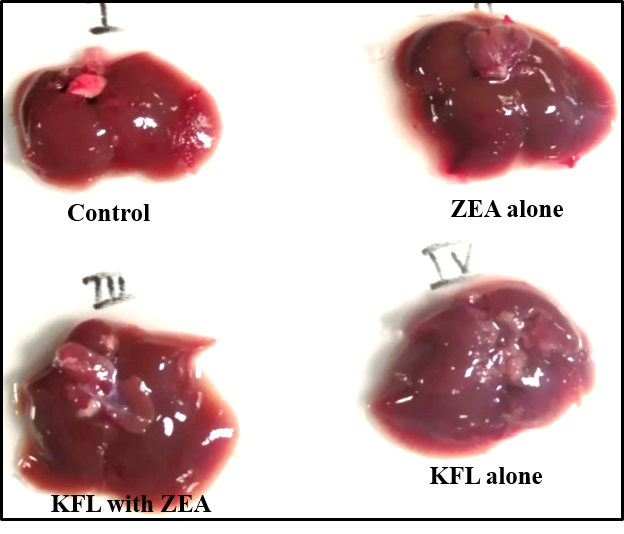


**Figure S2.** Liver morphology. Liver morphology there is no significance variance appearance
